# Early Identification of ST-Segment Elevation Myocardial Infarction (STEMI) at Presentation: Comparative Diagnostic Performance of CBC-Derived Inflammatory Indices and High-Sensitivity Troponin T

**DOI:** 10.3390/jcm15113998

**Published:** 2026-05-22

**Authors:** Chennet Phonphet, Putrada Ninla-aesong, Sasithorn Sanakus, Jom Suwanno, Ladda Thiamwong

**Affiliations:** 1 School of Nursing and the Excellent Center of Community Health Promotion, Walailak University, Nakhon Si Thammarat 80160, Thailand; pchennet@wu.ac.th (C.P.); sjom@wu.ac.th (J.S.); 2 School of Medicine and Research Center in Tropical Pathobiology, Walailak University, Nakhon Si Thammarat 80160, Thailand; 3 Maharaj Nakhon Si Thammarat Hospital, Nakhon Si Thammarat 80000, Thailand; junioryuita@gmail.com; 4 College of Nursing, University of Central Florida, Orlando, FL 32827, USA; ladda.thiamwong@ucf.edu

**Keywords:** acute coronary syndrome (ACS), ST-segment elevation myocardial infarction (STEMI), high-sensitivity troponin T (hs-Troponin T), complete blood count (CBC), neutrophil-to-lymphocyte ratio (NLR), neutrophil-to-lymphocyte × platelet ratio (NLPR), systemic inflammatory indices, early diagnosis, first medical contact, biomarkers

## Abstract

**Background/Objectives:** Early identification of ST-segment elevation myocardial infarction (STEMI) at first medical contact remains challenging, as high-sensitivity troponin T may be insufficiently sensitive during the initial phase of myocardial injury. Readily available complete blood count (CBC)-derived inflammatory indices may provide complementary early diagnostic signals. This study aimed to evaluate whether baseline CBC-derived inflammatory indices differ between STEMI and NSTEMI and whether they provide adjunctive discriminatory information at presentation (0 h) in patients with acute coronary syndrome (ACS). **Methods:** A 12-lead electrocardiogram (ECG), high-sensitivity troponin T, and CBC were obtained at presentation from 252 patients with ACS (195 STEMI and 57 NSTEMI). Diagnostic performance was evaluated using receiver operating characteristic (ROC) curve analysis and 2 × 2 contingency tables to determine the area under the curve (AUC), sensitivity, specificity, positive predictive value (PPV), negative predictive value (NPV), and likelihood ratios. **Results:** High-sensitivity troponin T demonstrated the highest specificity (84.44%) and PPV (92.93%), supporting its role as a confirmatory biomarker; however, its low sensitivity (50.83%) and NPV (29.92%) may reduce its utility during early assessment. In contrast, WBC and neutrophil counts demonstrated relatively favorable discriminatory performance at presentation (AUC > 0.72; Youden’s index > 0.40). Among composite indices, NLPR demonstrated the highest sensitivity (88.66%) and NPV (53.19%), along with the lowest negative likelihood ratio (0.25), suggesting potential adjunctive value during early assessment. NLR, SII, SIRI, and adjusted NLR showed moderate performance, with aNLR providing a balanced sensitivity (67.01%) and specificity (74.55%). **Conclusions:** CBC-derived inflammatory indices, particularly neutrophil-based markers such as NLPR, may provide adjunctive discriminatory information during the early assessment of patients with ACS, particularly at first medical contact when baseline hs-Troponin T sensitivity may still be limited.

## 1. Introduction

Acute coronary syndrome (ACS) refers to a spectrum of clinical conditions caused by a sudden reduction in coronary blood flow. It remains one of the leading causes of death worldwide. ACS encompasses a spectrum of conditions, including unstable angina (UA), non-ST-segment elevation myocardial infarction (NSTEMI), and ST-segment elevation myocardial infarction (STEMI).

Myocardial necrosis is present, and cardiac biomarkers (e.g., troponin) are elevated in both NSTEMI and STEMI. Among these, STEMI represents a life-threatening emergency that requires immediate identification and reperfusion therapy to prevent irreversible myocardial damage and improve survival outcomes [1]. Therefore, timely and accurate differentiation between STEMI, NSTEMI, and UA is critical for guiding management and improving patient outcomes.

High-sensitivity cardiac Troponins are specific biomarkers for myocardial injury and represent the gold standard for diagnosing myocardial infarction (MI) [2]. High-sensitivity Troponin T (hs-Troponin T) is widely used in patients with ACS to detect myocardial injury, facilitate early MI diagnosis, and support risk stratification. Its high specificity supports its role as a reliable confirmatory biomarker for myocardial injury. However, at presentation (0 h), its sensitivity may be limited, particularly in the early phase of disease [3], highlighting the need for serial measurements and adjunctive biomarkers to improve early diagnostic performance.

Importantly, although cardiac troponins are the gold standard biomarkers for myocardial injury and the diagnosis of MI, the identification of STEMI is primarily based on 12-lead electrocardiogram (ECG) findings, which guide urgent reperfusion decisions at presentation.

The sensitivity of hs-Troponin T varies depending on the timing of presentation and patient population [2,4,5]. Circulating troponin levels typically begin to rise within 2–3 h after symptom onset. The levels will continue to rise until they reach a peak, typically at 12–48 h, and return to baseline within 4–10 days [6]. Consequently, during the early phase of MI, hs-Troponin levels may remain within normal range, particularly in patients without ST-segment elevation [7], resulting in false-negative findings and potentially delays in diagnosis. In addition, hs-Troponin may be elevated for conditions unrelated to ACS, such as sepsis, pulmonary embolism, chronic heart failure, chronic kidney disease, or critically ill patients [2,7]. These limitations underscore the need for adjunctive biomarkers, particularly those reflecting early inflammatory responses, to enhance diagnostic sensitivity at presentation while maintaining specificity.

Beyond mechanical obstruction, inflammation and plaque instability are central to the pathogenesis of ACS. Rupture or erosion of vulnerable atherosclerotic plaque triggers thrombus formation and subsequent myocardial injury. Dysregulated immune-inflammatory responses play a critical role in both plaque destabilization and thrombogenesis [8,9]. They also continue to influence outcomes after the acute event by promoting adverse remodeling, recurrent ischemic events, and increased long-term cardiovascular risk [10].

Elevated white blood cell (WBC) and neutrophil counts, along with reduced lymphocyte counts, have been associated with larger myocardial infarct size and worse clinical outcomes in patients with ACS [11,12,13,14]. Accordingly, complete blood count (CBC) derived ratios such as the neutrophil-to-lymphocyte ratio (NLR), monocyte-to-lymphocyte ratio (MLR) [15], and platelet-to-lymphocyte ratio (PLR) have been investigated as potential diagnostic markers of ACS [16].

More recently, novel systemic inflammatory indices—including systemic immune-inflammation index (SII), systemic inflammation response index (SIRI), aggregate index of systemic inflammation (AISI), and neutrophil-to-lymphocyte × platelet ratio (NLRP)—have been proposed as integrative markers that more comprehensively reflect immune-inflammatory activation than individual cell counts or simple ratios [14,17]. These indices incorporate key cellular components involved in vascular inflammation, endothelial injury, and thrombogenesis. Han et al. (2022) [17] further demonstrated that elevated SIRI was associated with greater disease severity and enhanced the prognostic performance of the GRACE risk score in ACS patients undergoing PCI.

Given that the pathophysiology of ACS involves both inflammation and thrombosis, composite indices integrating neutrophils (acute inflammation), lymphocytes (immune regulation), and platelets (thrombotic activity) may provide complementary inflammatory information beyond simpler two-component markers, particularly for early identification of STEMI at presentation.

Early identification of ST-segment elevation myocardial infarction (STEMI) at first medical contact remains challenging, particularly during the initial phase of myocardial injury when high-sensitivity troponin T may not yet be sufficiently elevated. Given their rapid availability and low cost, CBC-derived inflammatory indices may provide complementary adjunctive information during early assessment. However, the comparative diagnostic performance of these indices relative to hs-Troponin T at first medical contact (0 h) remains inadequately characterized.

Therefore, this study aimed to evaluate whether baseline CBC-derived inflammatory indices, particularly neutrophil-based indices, differ between STEMI and NSTEMI and whether they provide adjunctive discriminatory value for identifying STEMI at presentation (0 h) in patients with ACS. Diagnostic performance was assessed using sensitivity, specificity, positive predictive value (PPV), negative predictive value (NPV), and likelihood ratios.

## 2. Materials and Methods

### 2.1. Study Design and Setting

This retrospective, cross-sectional diagnostic study was conducted at Maharaj Nakhon Si Thammarat Hospital, a tertiary referral center for cardiovascular care in Nakhon Si Thammarat, Thailand. The study was conducted according to the guidelines of the Declaration of Helsinki and approved by the Ethics Committee of Walailak University, Thailand (protocol code: WUEC-25-179-01 and date of approval: 28 May 2025). As this study involved a retrospective review of de-identified data, the requirement for informed consent was waived.

### 2.2. Study Population and Sample Selection

The study population comprised patients diagnosed with ACS undergoing PCI during their index hospitalization between 1 October 2020 and 30 September 2021. The medical records of all patients admitted with a diagnosis of ACS undergoing PCI during the 12-month period were reviewed. All data were anonymized prior to analysis to ensure patient confidentiality.

The study population was restricted to ACS patients undergoing PCI to ensure diagnostic confirmation based on coronary angiography and comprehensive clinical evaluation.

### 2.3. Diagnosis and Classification of ACS

Patients were initially diagnosed with ACS and classified as UA, NSTEMI, or STEMI based on clinical presentation and ECG findings at first medical contact (0 h). Final diagnosis, group allocation, and the need for PCI were subsequently confirmed by a board-certified cardiologist using a comprehensive assessment of clinical presentation, serial cardiac biomarkers (hs-Troponin T), ECG evolution, and coronary angiographic findings, in accordance with the Fourth Universal Definition of Myocardial Infarction [18].

At first medical contact (0 h), STEMI was primarily identified based on characteristic ST-segment elevation on a 12-lead ECG, in accordance with guideline-recommended criteria, while hs-Troponin T was used to support the diagnosis of MI.

A 12-lead ECG, hs-Troponin T, and CBC were obtained at first medical contact (0 h) in the emergency department. Although serial assessments were performed as part of routine clinical care, the present analysis focused on baseline (first medical contact, 0 h) values.

### 2.4. Inclusion Criteria and Exclusion Criteria

Patients were included if they met all of the following:

Age > 18 years.

Final diagnosis of STEMI or NSTEMI based on clinical presentation, ECG findings, cardiac biomarkers, and coronary angiography.

Had complete clinical records and laboratory data, including baseline 12-lead ECG, hs-Troponin T, and CBC results obtained at presentation (first medical contact, 0 h).

The study exclusion criteria were as follows:

Had any known hematologic malignancy or bone marrow disease, or were receiving immunosuppressive therapy.

Had active infection, sepsis, or a history of chronic inflammatory or autoimmune disease.

Undergone major surgery or experienced significant trauma within 30 days prior to hospitalization.

**Figure 1:**
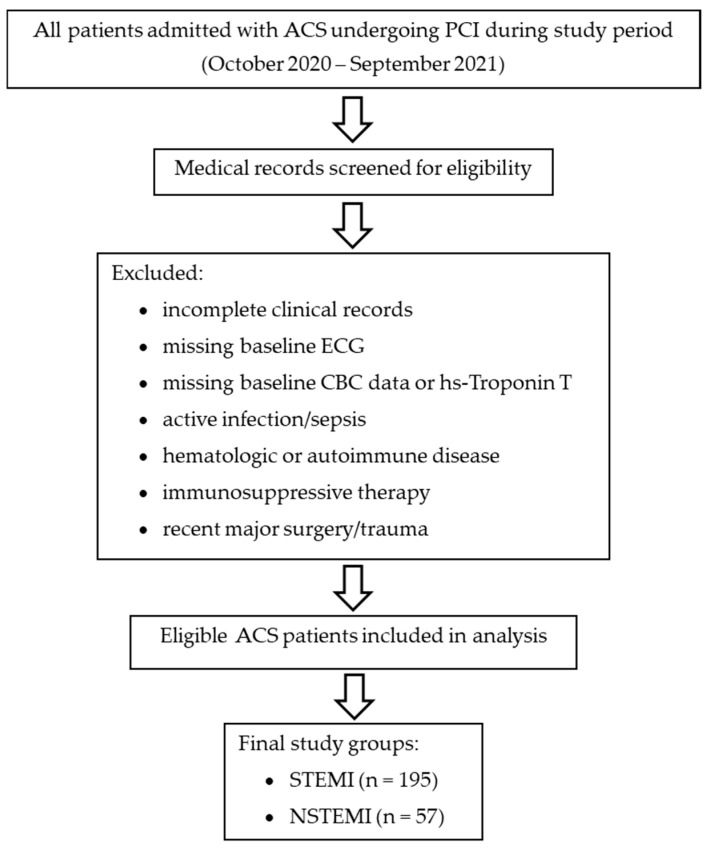
Flow chart of patient selection and study inclusion.

After applying these criteria, a total of 252 patients with ACS (195 STEMI and 57 NSTEMI) were included in the analysis. A flow chart summarizing patient selection, eligibility screening, exclusion criteria, and final study inclusion is presented in Figure 1.

### 2.5. Data Collection

Demographic data, clinical presentations, 12-lead ECG findings, hs-Troponin T, and CBC results obtained at first medical contact (0 h) in the emergency department, along with serial assessment data, were retrospectively extracted from patient medical records.

hs-Troponin T and CBC with differential were measured using routine commercial laboratory assays at the hospital laboratory. Based on CBC data, systemic inflammatory indices were calculated for each patient. The CBC-derivative inflammation indices included the NLR, MLR, PLR, SII, SIRI, AISI, NLPR, and adjusted NLR (aNLR, a novel inflammatory biomarker calculated as NLR × 100/platelet count).

### 2.6. Inflammatory Index Calculations

The CBC-derived systemic inflammatory markers were calculated as follows:

NLR: Neutrophil count/Lymphocyte count.

MLR: Monocyte count/Lymphocyte count.

PLR: Platelet count/Lymphocyte count.

SII: Neutrophil × Platelet/Lymphocyte.

SIRI: Neutrophil × Monocyte/Lymphocyte.

AISI: Neutrophil × Monocyte × Platelet/Lymphocyte.

NLPR: Neutrophil/(Lymphocyte × Platelet).

Adjusted NLR (aNLR): NLR × 100/Platelet.

All values were calculated using absolute counts (×10^12^ cells/m^3^).

### 2.7. Statistical Analysis

Continuous variables were reported as mean ± standard deviation (SD) or median (interquartile range [IQR]), depending on data distribution, and compared using the independent *t*-test or Mann–Whitney U test as appropriate. Categorical variables were presented as counts and percentages and compared using the Chi-square test or Fisher’s exact test, as appropriate. The diagnostic accuracy and clinical utility of each biomarker for confirming or ruling out STEMI were assessed using a range of statistical methods. The ability of each biomarker to predict STEMI in ACS patients was assessed using the Receiver Operating Characteristic (ROC) curve analysis. To evaluate overall discriminative ability, the area under the ROC curve (AUC) was computed. Youden’s Index (J = Sensitivity + Specificity − 1) was calculated across all possible thresholds to determine the optimal cut-off for each biomarker. The cut-off point that corresponded to the highest Youden’s Index was selected. Based on these optimal cut-offs, 2 × 2 contingency tables were constructed to determine sensitivity, specificity, positive predictive value (PPV), negative predictive value (NPV), and positive and negative likelihood ratios (LR^+^ and LR^−^). All statistical analyses were performed using SPSS (version 22, Chicago, IL, USA). A two-tailed *p*-value < 0.05 is considered statistically significant.

## 3. Results

Among a total of 252 ACS patients undergoing PCI included in this study, the majority were male (73.0%), with a mean age of 64.2 ± 12.7 years. The mean BMI of the study population was 23.44 ± 4.05 kg/m^2^, with 23.0% of patients classified as overweight (BMI ≥ 25 kg/m^2^) and 8.3% as obese (BMI ≥ 30 kg/m^2^). A history of smoking was reported in 57.2% of patients, with 39.3% being current smokers and 17.9% former smokers. Hypertension was the most prevalent comorbidity, present in 39.7% of patients, followed by dyslipidemia (28.2%) and type 2 diabetes mellitus (21.4%). A history of coronary artery disease and cerebrovascular disease was reported in 15.5% and 5.2% of patients, respectively. Fewer patients had other conditions such as chronic obstructive pulmonary disease (3.6%), chronic kidney disease (2.4%), asthma (1.6%), heart failure (0.8%), and cardiomegaly (0.8%). Overall, 36.9% of patients had at least two comorbidities. A total of 77.4% of patients were diagnosed with STEMI, and 22.6% with NSTEMI. The presumed underlying pathophysiological mechanism was atherosclerotic plaque rupture or erosion in 90.9% of cases, whereas thrombotic occlusion was identified in 9.1%. Additional angiographic and procedural characteristics, including culprit vessel distribution, STEMI wall/location, and number of stents used, are provided in Appendix A Table A1.

Table 1 shows the baseline characteristics of patients with ACS undergoing PCI, comparing those with STEMI and those with NSTEMI. Of the 252 patients undergoing PCI, 195 (77.4%) were diagnosed with STEMI, and 57 (22.6%) with NSTEMI. A significantly higher proportion of STEMI patients were male (76.9% vs. 59.6%, *p* = 0.010), and the mean age was slightly older in the STEMI group compared with the NSTEMI group (65.8 ± 12.6 vs. 62.4 ± 12.5 years, *p* = 0.034). Body mass index (BMI) was similar between the groups. Smoking status differed significantly (*p* = 0.002), with current smoking being more prevalent in STEMI patients (45.1%) compared with the NSTEMI group (19.3%).

Regarding comorbidities, dyslipidemia (40.4% vs. 24.6%, *p* = 0.020), coronary artery disease (26.3% vs. 12.3%, *p* = 0.010), and asthma (5.3% vs. 0.5%, *p* = 0.012) were more prevalent in the NSTEMI group. Other comorbidities, such as hypertension, diabetes mellitus, and chronic kidney disease, were similar between the groups. The mean number of comorbidities did not differ significantly between groups (1.2 ± 1.4 vs. 1.2 ± 1.2, *p* = 0.921).

The underlying cause of ACS also differed significantly (*p* = 0.007); atherosclerotic plaque rupture or erosion accounted for all NSTEMI cases, whereas thrombotic occlusion was identified in 11.8% of STEMI cases. The average length of hospital stay was longer in STEMI patients (3.67 ± 3.45 days) compared with NSTEMI patients (2.69 ± 2.22 days, *p* = 0.003) (Table 1).

Table 2 shows baseline (first medical contact, 0 h) CBC parameters, inflammatory markers, and hs- Troponin T in ACS patients undergoing PCI, comparing STEMI and NSTEMI groups. hs-Troponin T was significantly higher in the STEMI group compared with NSTEMI (2086.0 ± 5781.4 ng/L vs. 321.9 ± 472.9 ng/L, *p* ≤ 0.0001). Inflammatory cell differentials varied between groups: STEMI patients had higher neutrophil percentages (73.47% vs. 65.87%, *p* = 0.001) and lower lymphocyte (17.56% vs. 23.22%, *p* < 0.0001) and monocyte percentages (5.80% vs. 6.65%, *p* = 0.039). Eosinophil percentage was significantly lower in STEMI (1.48% vs. 3.58%, *p* < 0.0001), while basophil percentage was comparable.

Total white blood cell (WBC) count was significantly higher in STEMI patients (11.75 ± 4.50 × 10^12^ cells/m^3^) compared with NSTEMI patients (8.67 ± 3.18 × 10^12^ cells/m^3^, *p* < 0.0001). Similarly, absolute neutrophil counts were significantly higher in STEMI (8.81 ± 4.29 × 10^12^ cells/m^3^ vs. 5.87 ± 2.84 × 10^12^ cells/m^3^, *p* < 0.0001). Both eosinophil percentage and absolute eosinophil count were significantly lower in STEMI (1.48 ± 2.46% vs. 3.58 ± 4.02% and 0.15 ± 0.24 × 10^12^ cells/m^3^ vs. 0.30 ± 0.38 × 10^12^ cells/m^3^, respectively; *p* < 0.05). Platelet count tended to be lower in STEMI but did not reach statistical significance (Table 2).

Lymphocyte and monocyte parameters exhibited discordant changes in STEMI, with reduced proportions but unchanged or slightly increased absolute counts. Lymphocyte percentage was significantly decreased in STEMI (17.56 ± 10.53% vs. 23.22 ± 9.80%, respectively, *p*-value < 0.0001), while lymphocyte counts did not significantly differ (1.91 ± 1.20 × 10^12^ cells/m^3^ vs. 1.89 ± 0.84 × 10^12^ cells/m^3^, respectively, *p* = 0.893). Monocyte percentage was slightly decreased in STEMI (5.80 ± 2.31% vs. 6.65 ± 2.75%, respectively, *p* = 0.021), while monocyte counts were slightly increased (0.65 ± 0.31 × 10^12^ cells/m^3^ vs. 0.56 ± 0.27 × 10^12^ cells/m^3^, respectively, *p* = 0.044) (Table 2).

Regarding systemic inflammatory indices, STEMI patients demonstrated significantly higher NLR (6.49 ± 5.94 vs. 4.27 ± 4.71, *p* = 0.011), SIRI (4.70 ± 7.04 vs. 2.40 ± 3.30, *p* = 0.001), and aNLR (2.898 ± 3.314 vs. 1.689 ± 1.724, *p* < 0.0001). Other indices, including MLR and PLR, showed no statistically significant differences between the groups. SII, AISI, and NLPR tended to be higher in STEMI patients but did not reach statistical significance (*p* = 0.061, 0.065, and 0.061, respectively).

ROC curve analysis was conducted to evaluate the predictive value of baseline inflammatory markers and hs-Troponin T—the current gold standard biomarker for myocardial injury, particularly in the diagnosis of acute MI among ACS patients. Table 3 presents the area under the ROC curve (AUC) and optimal cut-off values for each biomarker. Among all parameters, WBC count demonstrated the highest discriminatory power (AUC = 0.732, 95% CI: 0.646–0.819, *p* < 0.001), followed closely by neutrophil count (AUC = 0.723, 95% CI: 0.639–0.808, *p* < 0.001) and hs-Troponin T (AUC = 0.711, 95% CI: 0.637–0.785, *p* < 0.001).

**Figure 2:**
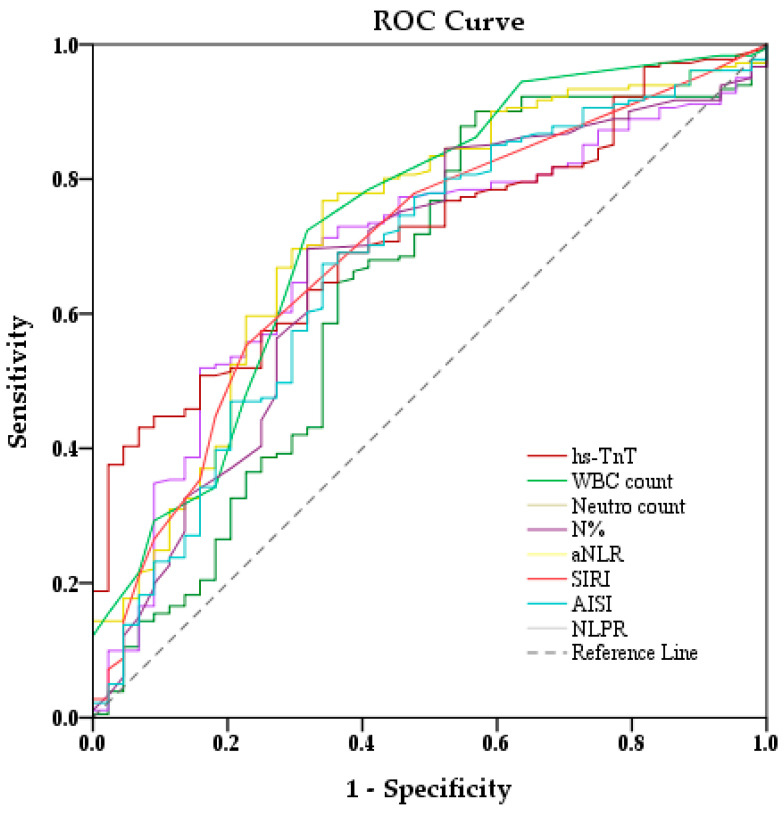
Receiver operating characteristic (ROC) curves of baseline inflammatory markers for identifying ST-segment elevation myocardial infarction (STEMI) among patients with acute coronary syndromes (ACS) undergoing percutaneous coronary intervention (PCI). ACS, acute coronary syndromes; PCI, percutaneous coronary intervention; STEMI, ST-segment elevation myocardial infarction; hs-TnT, high-sensitivity Troponin T; WBC, white blood cell; N%, neutrophil percentage; aNLR, adjusted neutrophil-to-lymphocyte ratio; SIRI, systemic inflammation response index; AISI, aggregate index of systemic inflammation; NLPR, neutrophil-to-lymphocyte × platelet ratio.

The optimal cut-off point for WBC count was >9.50 × 10^12^ cells/m^3^, yielding 72.4% sensitivity and 68.2% specificity. Neutrophil count > 6.08 × 10^12^ cells/m^3^ showed 76.2% sensitivity and 65.9% specificity. hs-Troponin T > 501.5 ng/L demonstrated high specificity (84.1%) but low sensitivity (50.8%) at presentation (Table 3 and Figure 2).

Other indices, including NLR, SII, SIRI, and aNLR, demonstrated moderate predictive performance, with AUCs of 0.668 (95% CI: 0.578–0.758), 0.642 (95% CI: 0.543–0.741), 0.695 (95% CI: 0.609–0.780), and 0.685 (95% CI: 0.601–0.769), respectively (all *p* < 0.05). aNLR > 1.5405 provided a balanced sensitivity of 70.2% and specificity of 68.2%, while SIRI > 2.50 showed a higher specificity of 77.3%. Monocyte count (AUC = 0.626, 95% CI: 0.534–0.717, *p* = 0.010) and AISI (AUC = 0.673, 95% CI: 0.582–0.764, *p* < 0.001) exhibited modest predictive value (Table 3).

Based on Youden’s Index, only the WBC count and neutrophil count demonstrated Youden’s Index values exceeding 0.40, indicating a more favorable balance between sensitivity and specificity and thus a more favorable sensitivity–specificity balance than baseline hs-Troponin T measurements at presentation. Among all biomarkers assessed, neutrophil count showed the highest Youden’s Index (0.421), reflecting the best overall diagnostic balance. Similarly, WBC count yielded a Youden’s Index of 0.406 at an optimal cut-off > 9.50 × 10^9^/L, with 72.4% sensitivity and 68.2% specificity. In contrast, although hs-Troponin T remains a widely accepted clinical biomarker, it showed a lower Youden’s Index (0.349), largely due to its relatively low sensitivity (50.8%) despite high specificity (84.1%). Meanwhile, MLR and monocyte count exhibited the lowest Youden’s Index values (0.216 and 0.188, respectively), indicating limited utility in diagnosing STEMI among ACS patients (Table 3).

Overall, the ROC analysis showed that the novel marker NLPR exhibited the highest sensitivity (90.1%) but had low specificity (40.9%), resulting in a limited Youden’s Index of 0.310. In contrast, hs-Troponin T had the highest specificity (84.1%) but lower sensitivity (50.8%) and a slightly higher Youden’s Index (0.349) (Table 3).

Table 4 summarizes diagnostic performances based on 2 × 2 contingency analyses using ROC-derived cut-offs. At >501.5 ng/L, hs-Troponin T yielded 50.83% sensitivity and 84.44% specificity. WBC count (>9.50) showed a balanced performance (69.59% sensitivity; 69.09% specificity). Neutrophil count (>6.08) slightly improved sensitivity (73.20%) with similar specificity. Monocyte count (>0.58) had a weaker diagnostic value (54.64% sensitivity; 60.00% specificity). Neutrophil percentage (N% > 72.5) offered a fair balance (65.98% sensitivity; 72.73% specificity).

Inflammatory indices such as NLR (>3.62) and SII (>949.5) showed moderate performance. SIRI (>2.50) had lower sensitivity (52.58%) but higher specificity (74.55%). AISI (>472.5) and MLR (>0.325) showed limited value. aNLR (>1.5405) demonstrated a good balance (67.01% sensitivity; 74.55% specificity). NLPR (>468.0) yielded the highest sensitivity (88.66%) but lowest specificity (45.45%), suggesting greater sensitivity for identifying STEMI at presentation, although specificity remained limited (Table 4).

Predictive values and likelihood ratios were also assessed. At their respective optimal cut-off points, hs-Troponin T had the highest PPV at 92.93%, followed by aNLR (90.28%), N% (89.51%), neutrophil count (89.31%), WBC count (88.82%), and SIRI (87.93%). NLPR also demonstrated a strong PPV of 85.15% despite its lower specificity (53.19%). In contrast, monocyte count and MLR had the lowest PPVs (82.81 and 81.82, respectively).

For NPV, NLPR (>468.0) showed the highest (53.19%), followed by neutrophil count (42.22%), WBC count (39.18%), and aNLR (39.05%). hs-Troponin T showed a low NPV of 29.92%**,** indicating the limitation of hs-Troponin T in ruling out STEMI in this context.

In likelihood ratio analysis, hs-Troponin T had the highest LR^+^ (3.27), followed by aNLR (2.63), neutrophil percentage (2.42), neutrophil count (2.37), WBC count (2.25), and SIRI (2.07). NLPR had the lowest LR^−^ (0.25), followed by neutrophil count (0.39), WBC count (0.44), aNLR (0.44), and N% (0.47), all outperforming hs-Troponin T (0.58) (Table 4).

These findings suggest that CBC-derived inflammatory indices, particularly neutrophil-based markers with high sensitivity, may serve as practical adjunctive tools for early risk stratification and clinical assessment at first medical contact, prior to definitive confirmation with hs-Troponin T.

## 4. Discussion

We hypothesized that composite inflammatory indices—particularly neutrophil-related markers such as NLR, SII, SIRI, AISI, aNLR, and NLPR—would differ between STEMI and NSTEMI and demonstrate adjunctive discriminatory value among patients with ACS, particularly at first medical contact (0 h), when the sensitivity of hs-Troponin T may still be limited. In this study of 252 ACS patients undergoing PCI, we systematically evaluated the diagnostic performance of baseline CBC-derived inflammatory biomarkers relative to hs-Troponin T in differentiating STEMI from NSTEMI at presentation.

Our findings demonstrate that several CBC-derived inflammatory indices, particularly neutrophil-based markers, demonstrated relatively favorable discriminatory performance at presentation in the acute setting of ACS, where rapid clinical decision-making is essential. At first medical contact, when hs-Troponin T levels may not yet be fully elevated, these readily available hematological parameters may provide adjunctive information that could support early clinical assessment and risk stratification in patients with ACS.

Consistent with known pathophysiology [14], patients with STEMI were more frequently male, older, and current smokers, and experienced more severe in-hospital complications compared with those with NSTEMI. The higher prevalence of smoking in STEMI (45.1% vs. 19.3%) supports its role as a potential acute trigger for thrombotic events. In contrast, chronic comorbidities such as dyslipidemia and coronary artery disease were more common in NSTEMI, suggesting a more progressive atherosclerotic process. These findings reinforce the concept that acute plaque rupture and thrombosis, rather than cumulative comorbidity burden alone, are key drivers of STEMI (Table 1).

In terms of inflammatory profiles, WBC and neutrophil counts, along with neutrophil-related indices (NLR, SIRI, and aNLR), were significantly elevated in STEMI, with trends toward higher SII, AISI, and NLPR values (Table 2). These observations are consistent with previous studies linking neutrophil-driven inflammation to ACS severity and adverse outcomes [11,12,13,14,15,19].

Although hs-Troponin T levels were significantly higher in STEMI at presentation (STEMI: 2086.0 ± 5781.4 vs. NSTEMI: 321.9 ± 472.9 ng/L, *p* < 0.0001), a wide variability and overlap in values between groups were observed. This likely reflects the temporal kinetics of troponin release, which may limit its sensitivity at the early phase of MI. Accordingly, while hs-Troponin T remains the gold standard biomarker for myocardial injury, our findings reinforce its role as a confirmatory biomarker for myocardial injury, while also highlighting the limitation of single baseline measurements during the early phase of presentation (Table 2).

Notably, WBC and neutrophil counts demonstrated relatively favorable discriminatory performance at presentation, with higher sensitivity and Youden’s Index values than baseline hs-Troponin T measurements, highlighting their potential as a simple and readily available adjunctive marker (Table 3). These findings may partly reflect the temporal limitation of a single baseline hs-Troponin T assessment during the early phase of myocardial injury. Our findings are consistent with previous reports linking elevated leukocyte and neutrophil counts to larger infarct size and worse clinical outcomes in patients with ACS [11,12,13,14,15,19]. In contrast, MLR and monocyte count demonstrated poor diagnostic performance, with the lowest Youden’s indices (0.216 and 0.188, respectively), indicating limited utility for differentiating STEMI from NSTEMI in this cohort.

Further analysis using 2 × 2 contingency tables and likelihood ratios provided additional insight into clinical applicability. hs-Troponin T demonstrated the strongest rule-in capability, reflected by the highest specificity and LR^+^ values, supporting its established role as a confirmatory biomarker for myocardial injury. However, its relatively low sensitivity and NPV at presentation may reflect the limitation of single baseline hs-Troponin T measurements during the early phase of myocardial injury. In contrast, NLPR showed the highest sensitivity and lowest negative likelihood ratio among the evaluated inflammatory indices, suggesting potential adjunctive value during the early assessment of patients with ACS (Table 4). However, given the non-specific nature of inflammatory biomarkers, these findings should be interpreted cautiously and within the context of established clinical and electrocardiographic assessment.

Other indices, including WBC count, neutrophil count, NLR, SII, SIRI, AISI, and aNLR, showed moderate discriminatory performance, whereas MLR and monocyte count demonstrated the lowest overall diagnostic value. aNLR performed well, with balanced sensitivity and specificity, moderate AUC, strong PPV, and an acceptable positive likelihood ratio, indicating its potential utility as a useful adjunctive biomarker to support early risk stratification (Table 4).

From a mechanistic perspective, the observed alterations in leukocyte subpopulations likely reflect neutrophil-driven expansion of the circulating leukocyte pool during acute inflammation. This results in relative reductions in lymphocyte and monocyte percentages despite preserved or slightly increased absolute counts.

Monocytes play a central role in post-infarction inflammation and tissue remodeling [10,20,21,22]. Their observed dynamics likely reflect mobilization from the bone marrow and recruitment to the injured myocardium, where they differentiate into macrophages and contribute to the local inflammatory response and cardiac repair following acute MI [21,23]. In addition, absolute eosinopenia was observed in STEMI patients, consistent with emerging evidence suggesting peripheral depletion due to eosinophil recruitment to the myocardium during the repair phase after MI [24]. The STEMI group also showed a trend toward lower platelet counts; however, this difference did not reach statistical significance.

The differences in diagnostic performance among CBC-derived inflammatory biomarkers may be explained by the mathematical structure of their components (SII: Neutrophil × Platelet/Lymphocyte; SIRI: Neutrophil × Monocyte/Lymphocyte; AISI: Neutrophil × Monocyte × Platelet/Lymphocyte; NLPR: Neutrophil/(Lymphocyte × Platelet); aNLR: NLR × 100/Platelet). Indices incorporating platelet counts in the denominator (e.g., NLPR, aNLR) may be amplified in the setting of reduced platelet levels, whereas those incorporating platelets in the numerator (e.g., SII) may be attenuated. Furthermore, indices derived from absolute cell counts (e.g., NLPR) may provide greater sensitivity to subtle changes, while normalized ratios (e.g., aNLR) may offer improved stability and balance between sensitivity and specificity.

Importantly, although a 12-lead electrocardiogram (ECG) remains the primary diagnostic tool for identifying STEMI, our findings suggest that CBC-derived inflammatory indices may serve as valuable adjunctive tools, particularly in early or equivocal presentations. In emergency department settings, where rapid decision-making is critical and laboratory turnaround time is essential, these readily available biomarkers may support early risk stratification before serial troponin measurements become informative.

## 5. Conclusions

hs-Troponin T demonstrates the highest specificity and PPV, supporting its role as a reliable confirmatory biomarker for STEMI. However, its limited sensitivity and low NPV at presentation may reduce its utility for early exclusion during the initial phase of myocardial injury. In contrast, several CBC-derived inflammatory indices, particularly neutrophil-based markers such as NLPR, demonstrated higher sensitivity and favorable sensitivity-based discriminatory characteristics.

These findings suggest that CBC-derived inflammatory indices may provide adjunctive discriminatory information between STEMI and NSTEMI at first medical contact among patients with ACS. Although these biomarkers are not intended to replace ECG or cardiac troponin testing, they may serve as readily available supportive markers for early risk stratification and clinical assessment in the acute setting.

## 6. Strengths and Limitations

This study has several strengths. First, it evaluates a comprehensive panel of CBC-derived inflammatory indices in a real-world cohort of ACS patients at first medical contact, providing clinically relevant insight into early diagnostic challenges. The use of multiple diagnostic metrics, including ROC analysis and likelihood ratios, further enhances the translational applicability of the findings. In addition, confounding was reduced by excluding patients with known hematologic malignancies, bone marrow disorders, active infections, recent significant trauma, or current immunosuppressive therapy, all of which are known to affect inflammatory markers.

However, this study also has limitations. CBC-derived inflammatory indices are biologically non-specific biomarkers and may be influenced by concurrent inflammatory or comorbid conditions. Therefore, although major inflammatory confounders were excluded, residual confounding from unmeasured conditions cannot be fully excluded. In addition, because this study included ACS patients undergoing PCI, the diagnostic performance observed in this cohort should be interpreted within the context of the study population and may warrant further validation in broader real-world populations presenting with suspected ACS.

## 7. Implications for Clinical Practice and Future Research

From a clinical perspective, CBC-derived inflammatory indices, particularly neutrophil-based markers, may serve as practical adjunctive tools in emergency settings where rapid laboratory turnaround is essential. Given that CBC testing is routinely available and rapidly processed, these indices may support early risk stratification alongside established electrocardiographic and cardiac biomarker assessment, particularly during the initial phase when baseline hs-Troponin T levels may still be limited.

Future studies should further investigate the potential adjunctive value of CBC-derived inflammatory indices alongside established electrocardiographic and cardiac biomarker assessments for early risk stratification in patients with ACS.

## Figures and Tables

**Table 1:** Baseline clinical characteristics of patients with ACS undergoing PCI.

	STEMI (*n* = 195)	NSTEMI (*n* = 57)	*p*-Value
Sex			0.010 *
Male	150 (76.9)	34 (59.6)	
Female	45 (23.1)	23 (40.4)	
Age			
Mean ± SD	65.8 ± 12.6	62.4 ± 12.5	0.034 *
BMI			
Mean ± SD	23.45 ± 4.15	23.43 ± 3.93	0.974
Smoker			0.002 *
Never	76 (39.0)	32 (56.1)	
Former	31 (15.9)	14 (24.6)	
Current	88 (45.1)	11 (19.3)	
Comorbidity (Yes)			
Hypertension	76 (39.0)	24 (42.1)	0.671
Dyslipidemia	48 (24.6)	23 (40.4)	0.020 *
Diabetes mellitus	38 (19.5)	16 (28.1)	0.165
Coronary artery disease	24 (12.3)	15 (26.3)	0.010 *
Cerebrovascular disease	12 (6.2)	1 (1.8)	0.187
Chronic obstructive pulmonary disease	6 (3.1)	3 (5.3)	0.434
Chronic kidney disease	3 (1.5)	3 (5.3)	0.105
Asthma	1 (0.5)	3 (5.3)	0.012 *
Heart failure	2 (1.0)	0 (0.0)	0.443
Cardiomegaly	2 (1.0)	0 (0.0)	0.443
Number of comorbidities	1.2 ± 1.2	1.2 ± 1.4	0.921
ACS characteristics			0.007 *
Atherosclerotic plaque rupture or erosion	172 (88.2)	57 (100.0)	
Thrombotic occlusion	23 (11.8)	0 (0.0)	
Length of hospital stay (days)	3.67 ± 3.45	2.69 ± 2.22	0.003 *

Values are n (%) or mean ± SD. * *p* < 0.05. ACS, acute coronary syndrome; PCI, percutaneous coronary intervention; BMI, body mass index; STEMI, ST-segment elevation myocardial infarction; NSTEMI, non–ST-segment elevation myocardial infarction.

**Table 2:** Baseline complete blood count (CBC) parameters, inflammatory markers, and hs-Troponin T at presentation (0 h) in patients with ACS undergoing PCI.

Parameters	STEMI (*n*= 195)	NSTEMI (*n* = 57)	*p*-Value
hs-Troponin T (ng/L)	2086.0 ± 5781.4	321.9 ± 472.9	<0.0001 *
Neutrophil (%)	73.47 ± 14.94	65.87 ± 12.53	0.001 *
Lymphocyte (%)	17. 56 ± 10.53	23.22 ± 9.80	<0.0001 *
Monocyte (%)	5.80 ±2.31	6.65 ± 2.75	0.021 *
Basophil (%)	0.45 ± 0.74	0.47 ± 0.54	0.818
Eosinophil (%)	1.48 ± 2.46	3.58 ± 4.02	<0.0001 *
WBC count (10^12^ cells/m^3^)	11.75 ± 4.50	8.67 ± 3.18	<0.0001 *
Platelet count (10^12^ cells/m^3^)	245.90 ± 67.43	250.20 ± 73.15	0.682
Neutrophil (10^12^ cells/m^3^)	8.81 ± 4.29	5.87 ± 2.84	<0.0001 *
Lymphocyte (10^12^ cells/m^3^)	1.91 ± 1.20	1.89 ± 0.84	0.893
Monocyte (10^12^ cells/m^3^)	0.65 ± 0.31	0.56 ± 0.27	0.044 *
Eosinophil (10^12^ cells/m^3^)	0.15 ± 0.24	0.30 ± 0.38	0.008 *
NLR	6.49 ± 5.94	4.27 ± 4.71	0.011 *
MLR	0.48 ± 0.54	0.36 ± 0.28	0.118
PLR	173.75 ± 120.05	174.67 ± 154.89	0.963
SII	1596.06 ±1610.42	1147.51 ± 1368.66	0.061
SIRI	4.70 ± 7.04	2.40 ± 3.30	0.001 *
AISI	1202.04 ± 2073.12	667.33 ± 948.81	0.065
aNLR	2.898 ± 3.314	1.689 ± 1.724	<0.0001 *
NLPR	1596.06 ± 1610.42	1147.51 ± 1368.66	0.061

Data are shown as mean ± SD. * *p* < 0.05. ACS, acute coronary syndromes; PCI, percutaneous coronary intervention; STEMI, ST-segment elevation myocardial infarction; NSTEMI, non–ST-segment elevation myocardial infarction; WBC, white blood cell; NLR, neutrophil-to-lymphocyte ratio; PLR, platelet-to-lymphocyte ratio; SII, systemic immune-inflammatory index; SIRI, systemic inflammation response index; AISI, aggregate index of systemic inflammation; NLPR, neutrophil-to-lymphocyte × platelet ratio; and aNLR, adjusted NLR.

**Table 3:** Area under the receiver operating characteristic (ROC) curve and optimal cut-off values of baseline inflammatory markers for identifying ST-segment elevation myocardial infarction (STEMI) in patients with ACS undergoing PCI.

Biomarkers	AUC	95% CI of AUC	*p*-Value	Cut-Off Point	Sensitivity (%)	Specificity (%)	Youden’s Index
hs-Troponin T	0.711	0.637–0.785	0.000 *	>501.5	50.8	84.1	0.349
WBC count	0.732	0.646–0.819	0.000 *	>9.50	72.4	68.2	0.406
Neutrophil count	0.723	0.639–0.808	0.000 *	>6.08	76.2	65.9	0.421
Monocyte count	0.626	0.534–0.717	0.010 *	>0.58	55.2	63.6	0.188
% Neutrophil	0.672	0.581–0.762	0.000 *	>72.5	69.6	68.2	0.378
NLR	0.668	0.578–0.758	0.001 *	>3.62	69.6	63.6	0.332
MLR	0.597	0.511–0.684	0.046 *	>0.325	58	63.6	0.216
SII	0.642	0.543–0.741	0.003 *	>949.5	64.6	63.6	0.282
SIRI	0.695	0.609–0.780	0.000 *	>2.50	55.2	77.3	0.325
AISI	0.673	0.582–0.764	0.000 *	>472.5	67.4	65.9	0.333
aNLR	0.685	0.601–0.769	0.000 *	>1.5405	70.2	68.2	0.384
NLPR	0.642	0.543–0.741	0.003 *	>468.0	90.1	40.9	0.310

* *p* < 0.05. ACS, acute coronary syndromes; PCI, percutaneous coronary intervention; STEMI, ST-segment elevation myocardial infarction; 95% CI, 95% confidence intervals; AUC, area under the receiver operating characteristic (ROC) curve; hs-Troponin T, high-sensitivity Troponin T; WBC, white blood cell; NLR, neutrophil-to-lymphocyte ratio; SII, systemic immune-inflammatory index; SIRI, systemic inflammation response index; AISI, aggregate index of systemic inflammation; NLPR, neutrophil-to-lymphocyte × platelet ratio; and aNLR, adjusted NLR.

**Table 4:** Diagnostic performance of selected baseline biomarkers for identifying STEMI in patients with ACS undergoing PCI, based on 2 × 2 contingency analysis using ROC-derived cut-off values.

Biomarkers	Cut-Off Point	Sensitivity (%)	Specificity (%)	PPV (%)	NPV (%)	Chi-Square *p*-Value	LR^+^	LR^−^
hs-Troponin T	>501.5	50.83	84.44	92.93	29.92	0.000 *	3.27	0.58
WBC count	>9.50	69.59	69.09	88.82	39.18	0.000 *	2.25	0.44
Neutrophil count	>6.08	73.20	69.09	89.31	42.22	0.000 *	2.37	0.39
Monocyte count	>0.58	54.64	60.00	82.81	27.27	0.039 *	1.37	0.76
% Neutrophil	>72.5	65.98	72.73	89.51	37.74	0.000 *	2.42	0.47
NLR	>3.62	65.98	63.64	86.49	34.65	0.000 *	1.81	0.53
MLR	>0.325	55.67	56.36	81.82	26.50	0.077	1.28	0.79
SII	>949.5	61.34	63.64	85.61	31.82	0.001 *	1.69	0.61
SIRI	>2.50	52.58	74.55	87.93	30.83	0.000 *	2.07	0.64
AISI	>472.5	64.95	65.45	86.90	34.62	0.000 *	1.88	0.54
aNLR	>1.5405	67.01	74.55	90.28	39.05	0.000 *	2.63	0.44
NLPR	>468.0	88.66	45.45	85.15	53.19	0.000 *	1.63	0.25

* *p* < 0.05. ACS, acute coronary syndromes; PCI, percutaneous coronary intervention; STEMI, ST-segment elevation myocardial infarction; PPV, Positive predictive value; NPV, Negative predictive value; LR^+^, Positive likelihood ratio; LR^−^, Negative likelihood ratio; hs-Troponin T, high-sensitivity Troponin T; WBC, white blood cells; NLR, neutrophil-to-lymphocyte ratio; SII, systemic immune-inflammatory index; SIRI, systemic inflammation response index; AISI, aggregate index of systemic inflammation; NLPR, neutrophil-to-lymphocyte × platelet ratio; and aNLR, adjusted NLR.

## Data Availability

The raw data supporting the conclusions of this article will be made available by the authors on request.

## Abbreviations

The following abbreviations are used in this manuscript:

ACS	Acute coronary syndromes
UA	Unstable angina
STEMI	ST-segment elevation myocardial infarction
NSTEMI	Non-ST-segment elevation myocardial infarction
PCI	Percutaneous coronary intervention
hs-Troponin T	High-sensitivity Troponin T
CBC	Complete blood count
WBC	White blood cell
NLR	Neutrophil-to-lymphocyte ratio
PLR	Platelet-to-lymphocyte ratio
SII	Systemic immune-inflammatory index
SIRI	Systemic inflammation response index
AISI	Aggregate index of systemic inflammation
NLPR	Neutrophil-to-lymphocyte × platelet ratio
aNLR	Adjusted NLR
BMI	Body mass index
ROC curves	Receiver operating characteristic (ROC) curves
AUC	Area under the ROC curve
95% CI	95% confidence intervals
mean ± SD	Mean ± standard deviation

## Appendix A

**Table A1:** Characteristics of patients with acute coronary syndromes (ACS) undergoing percutaneous coronary intervention (PCI).

Characteristics	Population (n = 252)
Sex	
Male	184 (73.0)
Female	68 (27.0)
Age	
Mean ± SD	64.2 ± 12.7
BMI	
Mean ± SD	23.44 ± 4.05
BMI group	
<18.5	24 (9.5)
18.5–22.9	99 (39.3)
23.0–24.9	50 (19.9)
25.0–29.9	58 (23.0)
>30.0	21 (8.3)
Diagnosis	
UA	0 (0)
NSTEMI	57 (22.6)
STEMI	195 (77.4)
Smoker	
Never	108 (42.8)
Former	45 (17.9)
Current	99 (39.3)
Comorbidity	
Hypertension	100 (39.7)
Dyslipidemia	71 (28.2)
Diabetes mellitus	54 (21.4)
Coronary artery disease	39 (15.5)
Cerebrovascular disease	13 (5.2)
Chronic obstructive pulmonary disease	9 (3.6)
Chronic kidney disease	6 (2.4)
Asthma	4 (1.6)
Heart failure	2 (0.8)
Cardiomegaly	2 (0.8)
Having at least two concurrent conditions	93 (36.9)
Cause of ACS	
Atherosclerotic plaque rupture or erosion	229 (90.9)
Thrombotic occlusion	23 (9.1)
Angiographic and procedural characteristics	
Culprit vessel, n (%)	
RCA/RPAD/RPL	89 (35.3)
LM	10 (4.0)
LAD/DG	131 (52.0)
LCx/OM	22 (8.7)
STEMI wall/location, n (%) *	
Anterior	99 (57.2)
Inferior	72 (41.6)
Posterior	1 (0.6)
Lateral	1 (0.6)
Number of stents used, median (IQR)	1 (1–2)

Values are n (%) or mean ± SD. * Percentages were calculated based on patients with available data. STEMI wall/location data were unavailable in 22 STEMI patients. ACS, acute coronary syndrome; PCI, percutaneous coronary intervention; BMI, body mass index; UA, unstable angina; STEMI, ST-segment elevation myocardial infarction; NSTEMI, non-ST-segment elevation myocardial infarction; RCA, right coronary artery; RPDA, right posterior descending artery; RPL, right posterolateral artery; LM, left main coronary artery; LAD, left anterior descending artery; DG, diagonal branches (arteries branch off the LAD); LCx, left circumflex artery; OM, obtuse marginal branches (branching vessels that emerge from the LCx); IQR, interquartile range.

## References

[1] Kraler S., Mueller C., Libby P., Bhatt D.L. (2025). Acute coronary syndromes: Mechanisms, challenges, and new opportunities. Eur. Heart J..

[2] Krychtiuk K.A., Newby L.K. (2024). High-sensitivity cardiac troponin assays: Ready for prime time!. Annu. Rev. Med..

[3] Pickering J.W., Greenslade J.H., Cullen L., Flaws D., Parsonage W., George P., Worster A., Kavsak P.A., Than M.P. (2016). Validation of presentation and 3 h high-sensitivity troponin to rule-in and rule-out acute myocardial infarction. Heart.

[4] Arslan M., Dedic A., Boersma E., Dubois E.A. (2020). Serial high-sensitivity cardiac troponin T measurements to rule out acute myocardial infarction and a single high baseline measurement for swift rule-in: A systematic review and meta-analysis. Eur. Heart J. Acute Cardiovasc. Care.

[5] Haller P.M., Sörensen N.A., Hartikainen T.S., Goßling A., Lehmacher J., Toprak B., Twerenbold R., Richter J., Banko T., Korschid S. (2023). Rising and falling high-sensitivity cardiac troponin in diagnostic algorithms for patients with suspected myocardial infarction. J. Am. Heart Assoc..

[6] Kontos M.C., Turlington J.S. (2020). High-sensitivity troponins in cardiovascular disease. Curr. Cardiol. Rep..

[7] Lazar D.R., Lazar F.-L., Homorodean C., Cainap C., Focsan M., Cainap S., Olinic D.M. (2022). High-sensitivity troponin: A review on characteristics, assessment, and clinical implications. Dis. Markers.

[8] Nilsson L., Wieringa W.G., Pundziute G., Gjerde M., Engvall J., Swahn E., Jonasson L. (2014). Neutrophil/lymphocyte ratio is associated with non-calcified plaque burden in patients with coronary artery disease. PLoS ONE.

[9] Ramoni D., Carbone F., Kraler S., Di Vece D., Montecucco F., Liberale L. (2025). Inflammation-Driven Plaque Erosion in Atherosclerosis: A Focus on Complement System Pathways. Curr. Atheroscler. Rep..

[10] Wang X., Chen L., Wei J., Zheng H., Zhou N., Xu X., Deng X., Liu T., Zou Y. (2025). The immune system in cardiovascular diseases: From basic mechanisms to therapeutic implications. Signal Transduct. Target. Ther..

[11] Chia S., Nagurney J.T., Brown D.F., Raffel O.C., Bamberg F., Senatore F., Wackers F.J.T., Jang I.-K. (2009). Association of leukocyte and neutrophil counts with infarct size, left ventricular function and outcomes after percutaneous coronary intervention for ST-elevation myocardial infarction. Am. J. Cardiol..

[12] Yalcinkaya E., Yuksel U.C., Celik M., Kabul H.K., Barcin C., Gokoglan Y., Yildirim E., Iyisoy A. (2015). Relationship between neutrophil-to-lymphocyte ratio and electrocardiographic ischemia grade in STEMI. Arq. Bras. Cardiol..

[13] Ferrari J.P., Lueneberg M.E., da Silva R.L., Fattah T., Gottschall C.A.M., Moreira D.M. (2016). Correlation between leukocyte count and infarct size in ST segment elevation myocardial infarction. Arch. Med. Sci. Atheroscler. Dis..

[14] Fan W., Wei C., Liu Y., Sun Q., Tian Y., Wang X., Liu J., Zhang Y., Sun L. (2022). The prognostic value of hematologic inflammatory markers in patients with acute coronary syndrome undergoing percutaneous coronary intervention. Clin. Appl. Thromb. Hemost..

[15] Shumilah A.M., Othman A.M., Al-Madhagi A.K. (2021). Accuracy of neutrophil to lymphocyte and monocyte to lymphocyte ratios as new inflammatory markers in acute coronary syndrome. BMC Cardiovasc. Disord..

[16] Pruc M., Peacock F.W., Rafique Z., Swieczkowski D., Kurek K., Tomaszewska M., Katipoglu B., Koselak M., Cander B., Szarpak L. (2022). The prognostic role of platelet-to-lymphocyte ratio in acute coronary syndromes: A systematic review and meta-analysis. J. Clin. Med..

[17] Han K., Shi D., Yang L., Wang Z., Li Y., Gao F., Liu Y., Ma X., Zhou Y. (2022). Prognostic value of systemic inflammatory response index in patients with acute coronary syndrome undergoing percutaneous coronary intervention. Ann. Med..

[18] Thygesen K., Alpert J.S., Jaffe A.S., Chaitman B.R., Bax J.J., Morrow D.A., White H.D., ESC Scientific Document Group (2019). Fourth universal definition of myocardial infarction (2018). Eur. Heart J..

[19] Dziedzic E.A., Gąsior J.S., Tuzimek A., Paleczny J., Junka A., Dąbrowski M., Jankowski P. (2021). Investigation of the associations of novel inflammatory biomarkers—Systemic inflammatory index (SII) and systemic inflammatory response index (SIRI)—With the severity of coronary artery disease and acute coronary syndrome occurrence. Int. J. Mol. Sci..

[20] Nah D.Y., Rhee M.Y. (2009). The inflammatory response and cardiac repair after myocardial infarction. Korean Circ. J..

[21] Peet C., Ivetic A., Bromage D.I., Shah A.M. (2020). Cardiac monocytes and macrophages after myocardial infarction. Cardiovasc. Res..

[22] Yap J., Irei J., Lozano-Gerona J., Vanapruks S., Bishop T., Boisvert W.A. (2023). Macrophages in cardiac remodelling after myocardial infarction. Nat. Rev. Cardiol..

[23] Mentkowski K.I., Euscher L.M., Patel A., Alevriadou B.R., Lang J.K. (2020). Monocyte recruitment and fate specification after myocardial infarction. Am. J. Physiol. Cell Physiol..

[24] Toor I.S., Rückerl D., Mair I., Ainsworth R., Meloni M., Spiroski A.-M., Benezech C., Felton J.M., Thomson A., Caporali A. (2020). Eosinophil deficiency promotes aberrant repair and adverse remodeling following acute myocardial infarction. J. Am. Coll. Cardiol. Basic Transl. Sci..

